# Diagnostic accuracy of clinical examination to identify life- and limb-threatening injuries in trauma patients

**DOI:** 10.1186/s13049-023-01083-z

**Published:** 2023-04-07

**Authors:** Jared M. Wohlgemut, Max E. R. Marsden, Rebecca S. Stoner, Erhan Pisirir, Evangelia Kyrimi, Gareth Grier, Michael Christian, Thomas Hurst, William Marsh, Nigel R. M. Tai, Zane B. Perkins

**Affiliations:** 1 Centre for Trauma Sciences, Blizard Institute, Queen Mary University of London, London, UK; 2 Ward 12D, Trauma Service, Royal London Hospital, Barts NHS Health Trust, Whitechapel Road, London, E1 1FR UK; 3 Academic Department of Military Surgery and Trauma, Royal Centre of Defence Medicine, Birmingham, UK; 4 Department of Electrical Engineering and Computer Science, Queen Mary University of London, London, UK; 5 London’s Air Ambulance, Royal London Hospital, Barts Health NHS Trust, London, UK

**Keywords:** Traumatic injuries, Diagnostic accuracy, Clinical examination, Uncertainty, Pre-hospital diagnosis

## Abstract

**Background:**

Timely and accurate identification of life- and limb-threatening injuries (LLTIs) is a fundamental objective of trauma care that directly informs triage and treatment decisions. However, the diagnostic accuracy of clinical examination to detect LLTIs is largely unknown, due to the risk of contamination from in-hospital diagnostics in existing studies. Our aim was to assess the diagnostic accuracy of initial clinical examination for detecting life- and limb-threatening injuries (LLTIs). Secondary aims were to identify factors associated with missed injury and overdiagnosis, and determine the impact of clinician uncertainty on diagnostic accuracy.

**Methods:**

Retrospective diagnostic accuracy study of consecutive adult (≥ 16 years) patients examined at the scene of injury by experienced trauma clinicians, and admitted to a Major Trauma Center between 01/01/2019 and 31/12/2020. Diagnoses of LLTIs made on contemporaneous clinical records were compared to hospital coded diagnoses. Diagnostic performance measures were calculated overall, and based on clinician uncertainty. Multivariate logistic regression analyses identified factors affecting missed injury and overdiagnosis.

**Results:**

Among 947 trauma patients, 821 were male (86.7%), median age was 31 years (range 16–89), 569 suffered blunt mechanisms (60.1%), and 522 (55.1%) sustained LLTIs. Overall, clinical examination had a moderate ability to detect LLTIs, which varied by body region: head (sensitivity 69.7%, positive predictive value (PPV) 59.1%), chest (sensitivity 58.7%, PPV 53.3%), abdomen (sensitivity 51.9%, PPV 30.7%), pelvis (sensitivity 23.5%, PPV 50.0%), and long bone fracture (sensitivity 69.9%, PPV 74.3%). Clinical examination poorly detected life-threatening thoracic (sensitivity 48.1%, PPV 13.0%) and abdominal (sensitivity 43.6%, PPV 20.0%) bleeding. Missed injury was more common in patients with polytrauma (OR 1.83, 95% CI 1.62–2.07) or shock (systolic blood pressure OR 0.993, 95% CI 0.988–0.998). Overdiagnosis was more common in shock (OR 0.991, 95% CI 0.986–0.995) or when clinicians were uncertain (OR 6.42, 95% CI 4.63–8.99). Uncertainty improved sensitivity but reduced PPV, impeding diagnostic precision.

**Conclusions:**

Clinical examination performed by experienced trauma clinicians has only a moderate ability to detect LLTIs. Clinicians must appreciate the limitations of clinical examination, and the impact of uncertainty, when making clinical decisions in trauma. This study provides impetus for diagnostic adjuncts and decision support systems in trauma.

**Supplementary Information:**

The online version contains supplementary material available at 10.1186/s13049-023-01083-z.

## Introduction

Timely and accurate identification of life- and limb-threatening injuries (LLTIs) is a fundamental objective of trauma care [1]. Identification of injuries directly informs triage and treatment decisions, therefore the accuracy of this action has a direct impact on patient outcomes [2–4]. In 1978, the American College of Surgeons Committee on Trauma proposed a structured clinical examination to enable life-threatening injuries to be identified and treated in a timely manner, and to reduce the risk of missed injuries [5]. This Advanced Trauma Life Support (ATLS) programme teaches a standardized method of initial trauma assessment and care, and has been adopted in over 80 countries worldwide [6] In addition to basic ATLS principles, a classification system of Injury Grading Scales for body regions and organs has been developed by the American Association for the Surgery of Trauma (AAST) which helps to define LLTIs [7–10].

Pre-hospital clinicians are not expected to identify all LLTIs. Indeed it may not be possible, as clinical examination may not elicit signs of certian injuries soon after trauma, making diagnosis difficult [11]. Modern pre-hospital physician-led trauma services have interventions to treat a large amount of life-threatening problems at their disposal. Many of these injuries are time-critical, and must be identified or highly suspected before initiating treatment. Delayed or missed identification of LLTIs is a leading cause of preventable morbidity and mortality [4, 12, 13]. For example, a study conducted in the 1980s found that, of 1000 consecutive injury-related deaths, nearly two thirds of non-central nervous system deaths were preventable, and these were principaly the result of missed injuries causing hemorrhage or hypoxia [13]. More recent analyses have reiterated that the majority of potentially preventable deaths after trauma are related to injury causing haemorrhage [14]. In contrast, overdiagnosis of LLTIs may expose patients to unnecessary interventions, impact the care of other patients (due to opportunity cost), and result in avoidable resource costs for the Trauma Center [15]. Therefore the topic of identifying LLTIs accurately is important, both for trauma patients and the systems which provide care.

The reported accuracy of clinical examination to identify LLTIs varies widely [16, 17], and precise estimates of diagnostic accuracy are largely unclear. This is because of an important limitation of existing diagnostic accuracy studies: in-hospital clinical examination findings may be contaminated by available diagnostic imaging results. Furthermore, it is often unclear how experienced the clinicians are who perform the examination. Known factors that influence the accuracy of clinical assessment in trauma include the mechanism of injury, polytrauma, and reduced level of consciousness [16–19]. Uncertainty is ubiquitous in emergency medical practice [20], yet its impact on the accuracy of clinical examination to identify LLTIs remains poorly understood.

The primary aim of this investigation was to determine the diagnostic accuracy of initial clinical examination, performed by expert trauma clinicians, to identify LLTIs. The secondary aims were to identify factors that impact the accuracy of clinical examination, in terms of missed identification and overdiagnosis of LLTIs, and determine the impact of clinician uncertainty on diagnostic accuracy.

## Methods

### Study design

This diagnostic accuracy study retrospectively reviewed consecutive adult trauma patients admitted to a single Major Trauma Centre (MTC), between 01/01/2019 and 31/12/2020. The study was reported according to the Standards for Reporting Diagnostic accuracy studies (STARD) (Additional file 1: Table S1) [21]. The study was approved by the institutional Clinical Effectiveness Unit (Registration number: 11736); research ethics committee review was waived.

### Study setting

The London Trauma System is the largest and busiest regional trauma system in the United Kingdom. In this system, an experienced pre-hospital trauma physician and paramedic are transported (by air or road) directly to seriously injured patients, to assess them for LLTIs and initiate resuscitation. Patients are then triaged and transported to the nearest appropriate hospital, which includes 35 trauma units and four MTCs. The trauma physician performs the patient examination. During the study period, no diagnostic adjuncts (including ultrasound) were available to the pre-hospital team.

Trauma physicians were experienced consultants (i.e. attendings) in either emergency medicine, anaesthesia, intensive care medicine or surgery (n = 20); or senior registrars (i.e. residents) with at least seven years post-graduate training (n = 4). All had significant experience of managing trauma patients both pre- and in-hospital, with a specialist interest in this field of medicine. Training is structured and rigorous, with simulation, workplace-based and didactic training.

### Selection of participants

Consecutive injured adult (≥ 16 years old) patients treated by London’s Air Ambulance (LAA) and admitted to one MTC were included. Patients were excluded if they were < 16 years old or suffered thermal injuries.

### Index test

Clinical examination findings were collected from contemporaneous pre-hospital records completed during the pre-hospital phase of care and handed over, together with the patient, to the receiving hospital trauma team. Clinicians are required to list suspected LLTIs on these records, and LAA conducts monthly audits to ensure form completion. Suspected injury diagnoses were compared to the reference standard. The level of certainty of pre-hospital diagnoses was also documented. As different clinicians use different words to describe their level of certainty, we based our categorisation of “certain” and “uncertain” on research conducted by the Central Intelligence Agency on how humans describe levels of probability [22]. Diagnoses were classified as having a high-level of certainty if documented with adjectives such as “likely”, “probably”, or without any qualifier [22]. Diagnoses were classified as having a low-level of certainty if documented with qualifying statements suggesting a low degree of certainty including “potentially”, “possibly”, “maybe”, “unlikely”, “rule out”, or “?” [22].

### Reference standard

Definitions of LLTIs were based on published classification systems, including the American Association for the Surgery of Trauma (AAST) Injury Grading Scales (Table 1) [7–10]. Definitive radiological, operative, and post-mortem findings were used as the reference standard. These findings were corroborated with data from the Trauma Audit and Research Network (TARN), an external prospective data registry that audits trauma performance [23]. Confirmed injuries were coded from source data, according to the Abbreviated Injury Scale (AIS).

**Table 1:** Classification of life- and limb-threatening injuries and bleeding [7–10]

Body region	Classification	Injuries
*Life-threatening injuries*		
Head	Mayo Classification (Moderate/Severe TBI)	Hematoma (Intracerebral, Subdural, or Epidural), Contusion (Cerebral or Hemorrhagic), Penetrating TBI (dura penetrated), Subarachnoid hemorrhage, Brain Stem Injury
Thorax	AAST Injury Scale ≥ 3	
Chest Wall		Flail Chest, Flail sternum, Avulsion of chest wall with underlying rib fractures
Heart		Blunt and penetrating cardiac injury, Cardiac tamponade, Avulsion of the heart
Haemothorax	NA	Hemothorax with > 20% blood loss, shocked (shock index > 0.9) or significant on-scene blood loss
Vascular		Arteries: Carotid, Innominate, Subclavian, Thoracic Aorta, Pulmonary (main trunk, and primary intraparenchymal branch) Veins: Superior and Inferior vena cava, Pulmonary (main trunk, and primary intraparenchymal branch)
Abdomen	AAST Injury Scale ≥ 3	
Liver		Any injury in the presence of a liver vascular injury or active bleeding; intraparenchymal laceration > 10 cm; ruptured liver hematoma; subcapsular hematoma > 50% surface area
Spleen		Any injury involving splenic vascular injury or active bleeding; parenchymal laceration > 3 cm depth; ruptured splenic hematoma; subcapsular hematoma > 50% surface area
Kidney		Any injury in the presence of a kidney vascular injury or active bleeding
Vascular		Arteries: Abdominal Aorta, Coeliac, Superior mesenteric, Renal, Iliac Veins: Inferior Vena Cava, Portal, Superior mesenteric, Renal, Iliac
Hollow organs		Bowel devascularisation, transection or disruption of > 50% of circumference Laceration (biliary, pancreas, extrahepatic bile duct), gallbladder avulsion, distal pancreas transection or parenchymal injury with duct injury, proximal pancreas transection or parenchymal injury involving ampulla, massive disruption of pancreatic head
Pelvis	AO/OTA classification (B/C)	B- Incomplete posterior arch disruption C- Complete posterior arch disruption
*Limb-threatening injuries*		
Spine		
Spinal Fracture	Denis	Any spinal fracture with 2 + spinous ligament injury, Chance fracture, Vertebral body < 50% loss, Hangman’s fracture, Jefferson’s fracture
Spinal cord	NA	Any Spinal cord injury
Extremity		
Long Bone Fracture	NA	Any Humerus, Radius, Ulna, Femur or Tibia fracture
Vascular	AAST Injury Scale ≥ 3	Arteries: Brachial, Axillary, Anterior tibial, Posterior tibial, Peroneal, Tibioperoneal trunk, Femoral (superficial, deep and common) Veins: Superficial or deep femoral, Popliteal
*Life-threatening bleeding*		
Thoracic Hemorrhage	AAST Injury Scale ≥ 4	Thoracic Vascular injury involving aorta, vena cava, or major pulmonary artery or vein. Cardiac injury with disruption of an atrium or ventricle Hemothorax with > 20% blood loss or shock (shock index > 0.9)
Abdominal Hemorrhage	AAST Injury Scale ≥ 4	Abdominal Vascular injury involving superior mesenteric artery, celiac axis, vena cava, aorta, portal vein injury, or any injury with > 20% blood loss Liver: Any injury in the presence of a liver vascular injury or active bleeding; parenchymal disruption > 25% of lobe Spleen: Any injury involving splenic vascular injury or active bleed Kidney: Any injury in the presence of a kidney vascular injury or active bleed

TBI, traumatic brain injury; AAST, The American Association of the Surgery for Trauma; AIS, Abbreviated Injury Scale; Hollow organs, stomach, duodenum, small bowel, colon, rectum, extrahepatic biliary or pancreas; AO, Arbeitsgemeinschaft für Osteosynthesefragen; OTA, Orthopaedic Trauma Association; tension pneumothorax is often treated pre-hospital, so is rarely present at time of arrival to hospital. It was therefore excluded, as the methodology compared accuracy of pre-hospital to hospital diagnosis; There was no AAST classification of hemothorax because it is not an injury but an anatomical state. We thus used a composite definition of major hemothorax

### Analyses

**Fig. 1:**
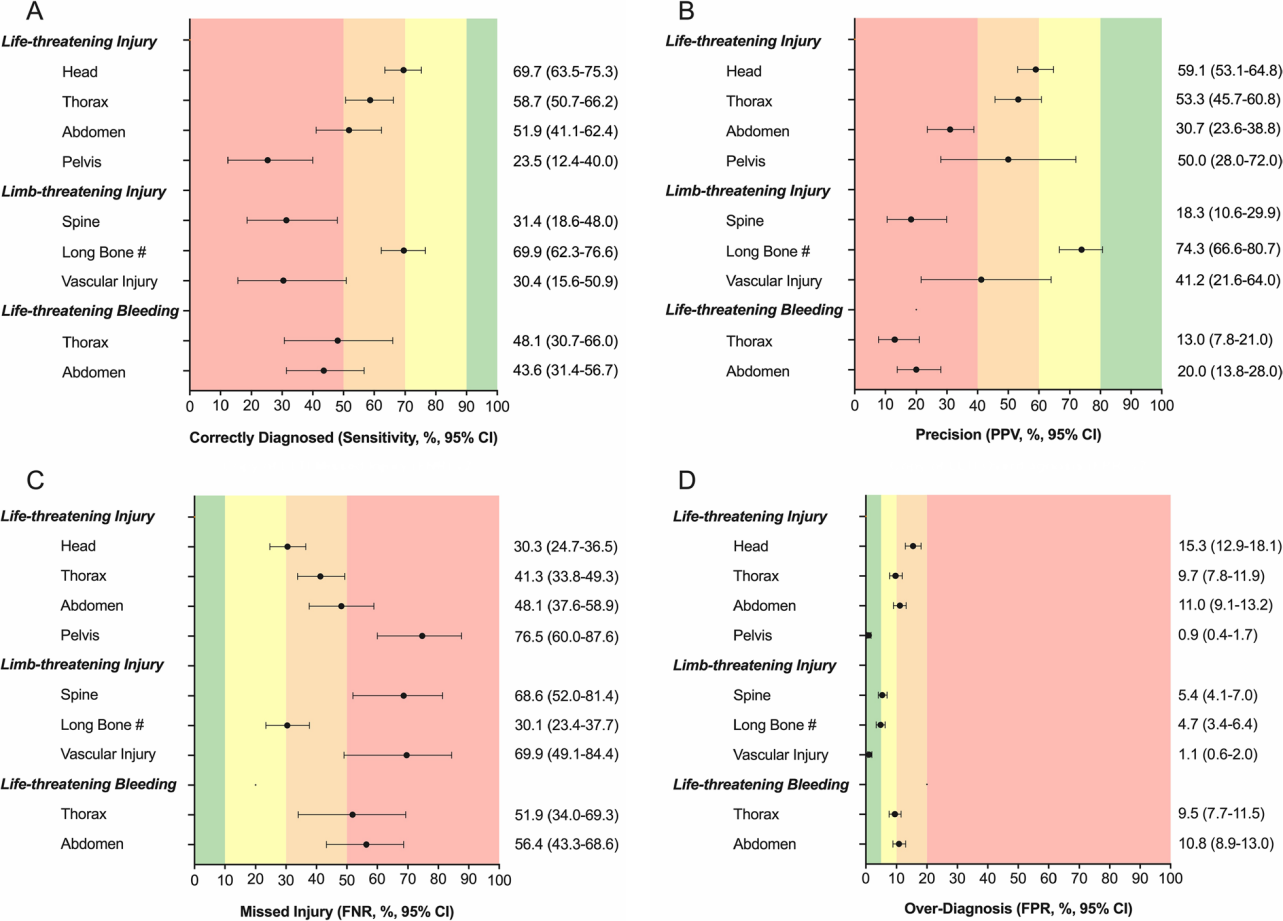
Diagnostic accuracy of clinical examination to identify life- and limb-threatening injuries and bleeding. Measures include **A** sensitivity, **B** Positive Predictive Value (PPV), **C** False Negative Rate (FNR) and **D** False Positive Rate (FPR). Black dots represent the accuracy measure, and horizontal lines represent 95% confidence intervals. Shaded vertical areas represent acceptable standards of accuracy measures

Analyses were conducted using Prism v9.0.2 (Graphpad Software Inc., San Diego, CA, USA). Data distribution was assessed using the D’Agostine & Pearson test. Continuous data were reported as medians with interquartile ranges (IQR) and categorical data as frequencies and percentages. Contingency tables were constructed, and standard measures of diagnostic performance were calculated: sensitivity, specificity, positive predictive value (PPV), negative predictive value (NPV), false positive rate (FPR; overdiagnosis), false negative rate (FNR; missed injuries), and likelihood ratio (LR), with 95% confidence intervals (CIs) [24]. Acceptable accuracy gradients (excellent, good, moderate, and poor) in the context of trauma resuscitation for sensitivity, PPV, FNR and FPR (Fig. 1) were agreed upon at a consensus meeting of LAA clinicians. Sensitivity analyses were performed according to the clinician’s degree of certainty of pre-hospital diagnoses. To explore risk factors for missed LLTIs, patient, clinical, and environmental factors were proposed a priori for inclusion in a univariate logistic regression model. Factors included: age, sex, mechanism of injury (MOI), Glasgow Coma Scale (GCS), systolic blood pressure (SBP), heart rate (HR), polytrauma (≥ 3 body regions injured according to AIS), base specialty of the treating clinician, shift pattern (day 06:45 to18:44; night 18:45 to 06:44), and clinician uncertainty. A similar model explored risk factors for overdiagnosis of LLTIs. Factors with a *p* value < 0.10 on univariate analysis were entered into a multivariable model while avoiding multi-collinearity using a forward stepwise method. Results were reported as odds ratios (ORs) with 95% CIs. Tests were 2-sided and *p* < 0.05 was considered significant.

## Results

### Characteristics of study participants

During the 2-year study period, LAA treated 3197 injured patients, of which 1042 were admitted to the MTC and were included in this study. Ninety-five patients were excluded: pediatric (82) and burns (13), leaving a study population of 947 patients. Their median age was 31 years (range: 16 to 89 years), 821 (86.7%) were male, and 569 (60.1%) suffered a blunt mechanism of injury (Table 2).

**Table 2:** Baseline characteristics of included patients (n = 947)

Characteristic	Missing no (%)	
Age, median (range), y	0	31 (16–89)
Sex	0	
Female		126 (13.3)
Male		821 (86.7)
Mechanism of injury	0	
Blunt		569 (60.1)
Penetrating		378 (39.9)
Injury	46 (4.9)	
ISS, median (IQR)		10 (4–22)
ISS > 15		364 (40.4)
AIS regions injured, median (IQR)		2 (1–3)
Polytrauma^a^		329 (36.6)
Life-threatening injury	0	
Head		228 (24.1)
Thorax		146 (15.4)
Abdomen		81 (8.6)
Pelvic Fracture		34 (3.6)
Limb-threatening injury	0	
Spinal Fracture		27 (2.9)
Long Bone Fracture		153 (16.1)
Vascular		23 (2.4)
Life-threatening bleeding	0	
Thorax		27 (2.9)
Abdomen		55 (5.8)
Pre-hospital Physiology		
GCS, median (IQR)	7 (0.7)	15 (10–15)
SBP, median (IQR), mm Hg	113 (11.9)	127 (109–143)
HR, median (IQR), beats/min	10 (1.1)	93 (79–109)
RR, median (IQR), breaths/min	12 (1.3)	18 (14–20)
Outcome		
In-hospital death	0	79 (8.3)
Hospital LOS, median (IQR), d	35 (3.7)	5 (2–18)
ICU LOS, median (IQR), d	35 (3.7)	0 (0–1)

Data presented as frequency (%), unless otherwise stated. Patients included all adult (≥ 16) patients assessed by London Air Ambulance and admitted to the Major Trauma Centre, 2019–2020

^a^Polytrauma, multiply injured patients, defined as ≥ 3 AIS body regions injured; PH, pre-hospital; ISS, injury severity score; AIS, abbreviated injury scale; GCS, Glasgow Coma Score; SBP, systolic blood pressure; HR, heart rate; RR, respiratory rate; IQR, inter-quartile range; LOS, length of hospital stay; ICU, intensive care unit; Dayshift was defined as between 06:45–18:44; Nightshift was defined as between 18:45–06:44

### Main results

#### Diagnostic accuracy of clinical examination

Of the 947 patients, 413 (43.6%) had a life-threatening injury, 188 (19.9%) had a limb-threatening injury, and 76 (8.0%) had life-threatening torso hemorrhage. The accuracy of diagnosing LLTIs is reported below (Fig. 1; Additional file 1: Table S2). The accuracy of diagnosing any injury is reported in the Supplementary Digital Content (Additional file 1: Table S3).

#### Life-threatening injury

Three hundred seventy-three patients (39.4%) suffered head injuries, of which 228 (61.1%) were classified as life-threatening. Life-threatening head injuries were correctly diagnosed in 159 patients (sensitivity 69.7%), missed in 69 patients (FNR 30.3%), and incorrectly diagnosed in 110 patients (FPR 15.3%). The likelihood of a patient being diagnosed with a life-threatening head injury actually having one was 59.1% (Fig. 1).

Four hundred and five patients (42.8%) suffered thoracic injuries, of which 146 (36.0%) were classified as life-threatening. Life-threatening thoracic injuries were correctly diagnosed in 87 patients (sensitivity 58.7%), missed in 59 patients (FNR 41.3%), and incorrectly diagnosed in 78 patients (FPR 9.7%). The likelihood of a patient being diagnosed with a life-threatening thoracic injury actually having one was 53.3% (Fig. 1).

Two hundred and forty-nine patients (26.3%) suffered abdominal injuries, of which 81 (32.5%) were classified as life-threatening. Life-threatening abdominal injuries were correctly diagnosed in 42 patients (sensitivity 51.9%), missed in 39 patients (FNR 48.1%), and incorrectly diagnosed in 95 patients (FPR 11.0%). The likelihood of a patient being diagnosed with a life-threatening abdominal injury actually having one was 30.7% (Fig. 1).

Eighty-six patients (9.1%) suffered a pelvic fracture, of whom 34 (39.5%) had unstable fractures. Unstable pelvic fractures were correctly diagnosed in eight patients (sensitivity 23.5%), missed in 26 patients (FNR 76.5%), and incorrectly diagnosed in eight patients (FPR 0.9%). The likelihood of a patient being diagnosed with an unstable pelvic fracture actually having one was 50.0% (Fig. 1).

#### Limb-threatening injury

One hundred and sixty-nine patients (17.8%) suffered spinal injuries, of which 27 (16.0%) were unstable spine fractures. Unstable spinal fractures were identified in 4 patients (sensitivity 14.8%), not identified in 23 patients (FNR 85.2%), and incorrectly diagnosed in 48 patients (FPR 5.2%). The likelihood of a patient being diagnosed with an unstable spinal fracture actually having one was 7.7% (Fig. 1).

Four hundred and forty-five patients (47.0%) suffered extremity injuries, of which 153 (34.4%) were long bone fractures, and 23 (5.2%) were peripheral vascular injuries. Long bone fractures were correctly diagnosed in 107 patients (sensitivity 69.9%), missed in 46 patients (FNR 30.1%), and incorrectly diagnosed in 37 patients (FPR 4.7%). The likelihood of a patient being diagnosed with a long bone fracture actually having one was 74.3% (Fig. 1). Peripheral vascular injuries were correctly diagnosed in 7 patients (sensitivity 30.4%), missed in 16 patients (FNR 69.6%), and incorrectly diagnosed in 10 patients (FPR 1.1%). The likelihood of a patient being diagnosed with a peripheral vascular injury actually having one was 41.2% (Fig. 1).

#### Life-threatening bleeding

Twenty-seven patients had life-threatening thoracic bleeding and fifty-five patients had life-threatening abdominal bleeding. Life-threatening thoracic bleeding was correctly diagnosed in 13 patients (sensitivity 48.1%), missed in 14 patients (FNR 51.9%), and incorrectly diagnosed in 87 patients (FPR 9.5%). The likelihood of a patient being diagnosed with life-threatening thoracic bleeding actually having it was 13.0% (Fig. 1). Life-threatening abdominal bleeding was correctly diagnosed in 24 patients (sensitivity 43.6%), missed in 31 patients (FNR 56.4%), and incorrectly diagnosed in 96 patients (FPR 10.8%). The likelihood of a patient being diagnosed with life-threatening abdominal bleeding actually having it was 20.0% (Fig. 1).

### Factors influencing the diagnostic accuracy of clinical examination

Sex, MOI, polytrauma, GCS, SBP, and HR were associated with missed injuries on univariate analysis (*p* < 0.1; Table 3). After adjusting for confounding factors, polytrauma and shock (hypotension and tachycardia) remained significant independent risk factors for missed injury (Table 3). MOI, polytrauma, SBP, clinician base specialty, diagnostic uncertainty, and shift pattern were associated with overdiagnosis of injuries on univariate analysis (*p* < 0.1; Table 4). After adjusting for confounding factors, diagnostic uncertainty and shock (hypotension) remained significant independent risk factors for overdiagnosis of injuries (Table 4).

**Table 3:** Univariate and multivariate logistic regression analyses, with missed injuries as the dependent variable

Parameter	Missed injuries as dependent variable (n = 265)			
	Univariate		Multivariate	
	Odds ratio (95% CI)	*p*	Odds ratio (95% CI)	*p*
*Patient factors*				
Age	1.00 (0.991 to 1.01)	0.97		
Sex (male)	1.00			
Sex (female)	1.46 (0.973 to 2.16)	0.06	1.19 (0.743 to 1.87)	0.47
MOI (blunt)	1.00			
MOI (penetrating)	0.704 (0.522 to 0.944)	0.02	1.06 (0.724 to 1.54)	0.78
Not polytrauma	1.00			
Polytrauma	1.86 (1.66 to 2.08)	< 0.001	1.83 (1.62 to 2.07)	< 0.001
PH GCS	0.961 (0.929 to 0.994)	0.02	0.999 (0.957 to 1.04)	0.97
PH SBP	0.992 (0.988 to 0.997)	< 0.001	0.993 (0.988 to 0.998)	0.005
PH HR	1.01 (1.00 to 1.01)	0.006	1.01 (1.00 to 1.01)	0.03
*Clinician factors*				
Spec: Emergency Medicine	1.00			
Spec: Anesthesiology	1.14 (0.838 to 1.55)	0.4		
Spec: Intensive Care	1.04 (0.603 to 1.74)	0.89		
Diagnostic Certainty	1.00			
Diagnostic Uncertainty	0.921 (0.670 to 1.26)	0.621		
*Environment factors*				
Shift Pattern (Dayshift)	1.00			
Shift Pattern (Nightshift)	0.959 (0.722 to 1.28)	0.77		

The table shows missed life- and limb-threatening injuries (false negatives) as dependent variable. Model statistics represented are Odds Ratios (95% confidence intervals), *p* values

Referents in the model: for Female Sex was Male, for Penetrating MOI was Blunt, for Polytrauma was Not Polytrauma, for Anesthesiology and Intensive Care base specialty was Emergency Medicine, for Clinician Diagnostic Uncertainty was Clinician Diagnostic Certainty, and for Nightshift was Dayshift

MOI, mechanism of injury; GCS, Glasgow coma scale; SBP, systolic blood pressure; HR, heart rate; Spec, base specialty; Polytrauma, categorical variable meaning ≥ 3 body regions injuried according to abbreviated injury scale (AIS) categories

**Table 4:** Univariate and multivariate logistic regression analyses, with overdiagnosed injuries as the dependent variable

Parameter	Overdiagnosed injuries as dependent variable (n = 380)			
	Univariate		Multivariate	
	Odds ratio (95% CI)	*p*	Odds ratio (95% CI)	*p*
*Patient factors*				
Age	0.998 (0.990 to 1.01)	0.59		
Sex (male)	1.00			
Sex (female)	0.744 (0.498 to 1.10)	0.14		
MOI (blunt)	1.00			
MOI (penetrating)	0.777 (0.594 to 1.01)	0.06	0.940 (0.675 to 1.31)	0.72
Not polytrauma	1.00			
Polytrauma	1.18 (1.07 to 1.30)	< 0.001	1.12 (0.999 to 1.25)	0.05
PH GCS	0.991 (0.960 to 1.02)	0.57		
PH SBP	0.991 (0.987 to 0.995)	< 0.001	0.991 (0.986 to 0.995)	< 0.001
PH HR	1.0 (0.995 to 1.00)	0.88		
*Clinician factors*				
Spec: Emergency Medicine	1.00		1.00	
Spec: Anesthesiology	0.628 (0.470 to 0.836)	0.002	0.873 (0.627 to 1.21)	0.42
Spec: Intensive Care	0.568 (0.339 to 0.930)	0.03	0.707 (0.386 to 1.26)	0.25
Diagnostic Certainty	1.00			
Diagnostic Uncertainty	6.65 (4.89 to 9.12)	< 0.001	6.42 (4.63 to 8.99)	< 0.001
*Environment factors*				
Shift Pattern (Dayshift)	1.00			
Shift Pattern (Nightshift)	0.74 (0.570 to 0.960)	0.02	0.814 (0.600 to 1.10)	0.18

The table shows overdiagnosis (false positives) of life- and limb-threatening injuries on the right. Model statistics represented are Odds Ratios (95% confidence intervals), *p* values

Referents in the model: for Female Sex was Male, for Penetrating MOI was Blunt, for Polytrauma was Not Polytrauma, for Anesthesiology and Intensive Care base specialty was Emergency Medicine, for Clinician Diagnostic Uncertainty was Clinician Diagnostic Certainty, and for Nightshift was Dayshift

MOI, mechanism of injury; GCS, Glasgow coma scale; SBP, systolic blood pressure; HR, heart rate; Spec, base specialty; Polytrauma, categorical variable meaning ≥ 3 body regions injuried according to abbreviated injury scale (AIS) categories

### Impact of diagnostic uncertainty on diagnostic accuracy

**Fig. 2:**
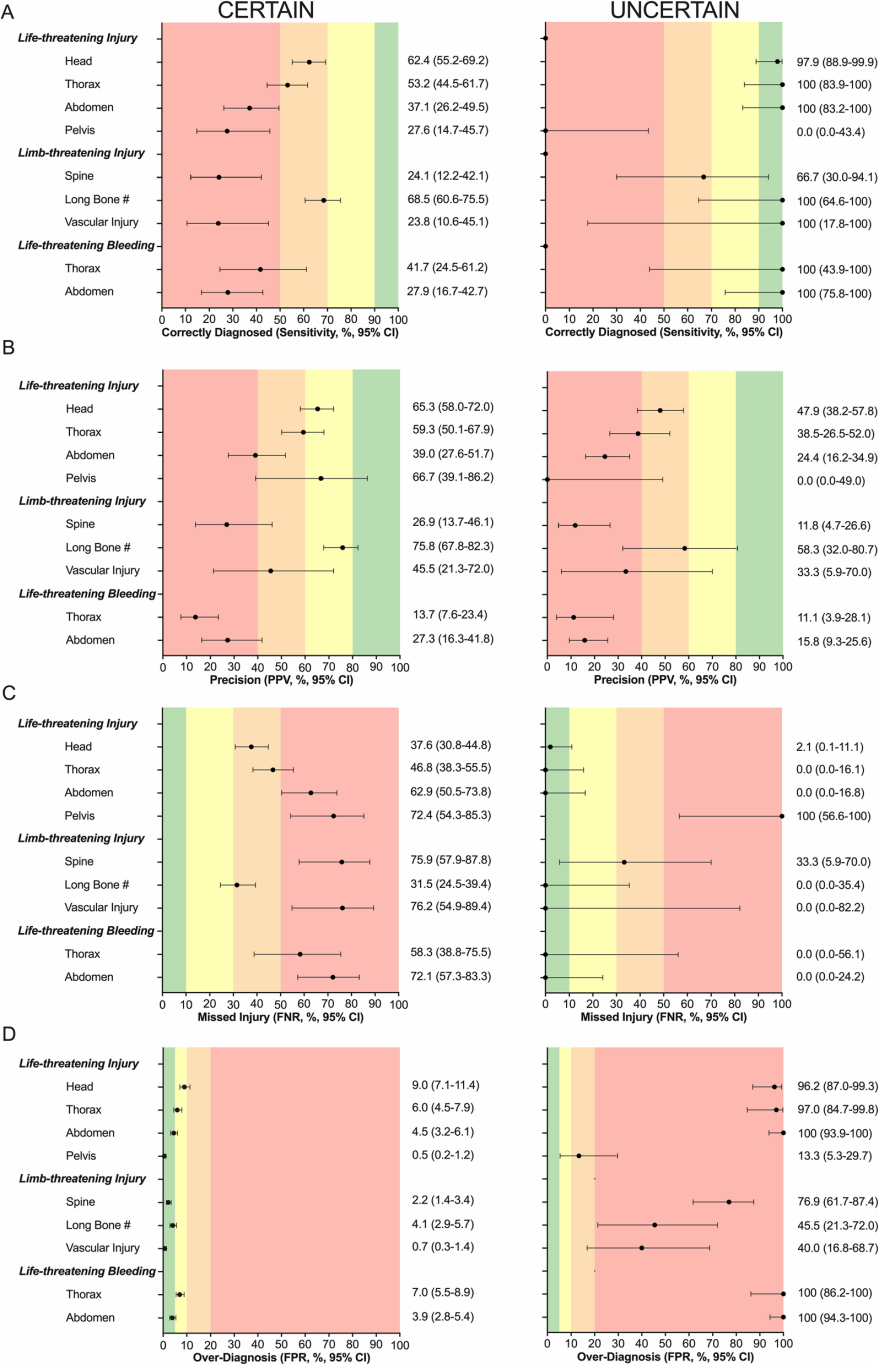
Diagnostic accuracy of clinical examination to identify life- and limb-threatening injuries and bleeding, according to clinician certainty. Measures include **A** sensitivity, **B** Positive Predictive Value (PPV), **C** False Negative Rate (FNR) and **D** False Positive Rate (FPR). Black dots represent clinician certainty, red dots uncertainty. Diagnoses were classified as having a high-level of certainty if documented with adjectives such as “likely”, “probably”, or without any qualifier. Diagnoses were classified as having a low-level of certainty if documented with qualifying statements suggesting a low degree of certainty including “potentially”, “possibly”, “maybe”, “unlikely”, “rule out”, or “?”. Horizontal lines represent 95% confidence intervals. Shaded vertical areas represent acceptable standards of accuracy measures

Diagnostic uncertainty was documented in 571 of 1517 (37.6%) LLTIs (Additional file 1: Table S4). The proportion of LLTI’s with diagnostic uncertainty varied by body region (highest for abdominal injuries and lowest for extremity injuries; Additional file 1: Tables S5 and S6). Overall, diagnostic uncertainty was associated with improved sensitivity but at the expense of a significant increase in overdiagnosis, with a resultant reduction in PPV (Fig. 2; Additional file 1: Tables S5 and S6).

## Discussion

The initial clinical examination of an injured patient, when performed by an experienced trauma clinician, had at best a moderate ability to identify life- or limb-threatening injuries, and major torso hemorrhage. The level of accuracy differed by body region, with clinical examination being more accurate for peripheral injuries than for torso injuries. Polytrauma and shock increased the chance of missed injuries, whereas shock and clinical uncertainty were associated with overdiagnosis. Diagnostic uncertainty was more common in torso injuries, and resulted in substantial overdiagnosis and, as a result, reduced precision.

The findings of this study have important implications for early (resuscitative) decision-making. In trauma, the purpose of clinical examination is to gather information, which is the first step in decision-making [25]. The next step is to understand the meaning of this information. A clear understanding of the accuracy of clinical examination is essential for understanding examination findings, enabling safe decision-making. Almost all decision-making errors made by experts are due to errors in these initial two-steps (gathering and understanding information), which are often termed errors in situational awareness [26–28]. Though not evaluated in this study, a clinician’s situational awareness may be influenced by mental workload and human factors such as stress and fatigue [28–31]. A challenge in the early assessment of trauma patients is that clinical signs may not yet be reliably present, predisposing clinicians to diagnostic and treatment errors. ATLS advises a number of adjuncts to the primary survey to improve diagnosis [5]. However, there are frequent situations where adjuncts are not immediately available and resuscitation decisions may rely on clinical findings. This study highlights the limitations of clinical examination in these situations.

Another implication of this study is that it highlights how difficult diagnosing non-compressivle torso haemorrhage (NCTH) is soon after injury, in regards to a high prevalence of clinical uncertainty and the low accuracy of clinical findings. NCTH is the leading cause of preventable deaths after injury [32]. Death from NCTH can be rapid, with most occurring within 6 h post-injury [33], and earlier intervention reduces mortality [34]. A recent major hemorrhage guideline acknowledged that “the major risks in emergency settings of bleeding remain delays in identification of the bleed, activation of major haemorrhage protocol (MHP) and timely provision of blood components” [35]. Therefore, clinicians should maintain a high index of suspicion of NCTH when the mechanism of injury is one that could result in life-threatening bleeding, even if clinical signs are not immediately apparent. Accordingly, pre-hospital time should be kept as short as possible, with the overarching goal of delivering a patient to a hospital that is capable of providing definitive hemorrhage control rapidly if required.

A further implication is the potential impact on the timeliness of diagnosis. Many key treatments, such as tranexamic acid (TXA), blood transfusion, and MHP activation, are most effective if provided as early as possible after injury [36–38]. These data demonstrate that early clinical examination has only a moderate ability to identify LLTIs and bleeding. Time is likely an important factor that influences accuracy; however waiting for LLTIs to become clinically obvious is an unsafe strategy. It may be safer to base therapeutic decisions on an overall risk assessment rather than relying on a definitive diagnosis. For example, TXA should be administered if there is a possibility of bleeding [36, 39] and the MHP should be activated when a patient is deemed at high risk for bleeding [35]. Nevertheless, clinicians’ lack of situational awareness of underlying injuries and bleeding may lead to delayed or missed opportunities for early intervention.

Accurate initial clinical assessment of trauma patients for LLTIs is a known challenge, especially for internal injuries of torso and major vascular injuries [11, 19, 40–48]. Studies have reported a wide range of sensitivity of clinical examination per body region: head (58–93%) [40, 42], thoracic (45–60%) [40, 42], abdominal (39–59%) [40, 42, 43], pelvic (45–86%) [19, 42, 44, 45], spinal (60–92%) [42, 46–48], and extremity injuries (33–91%) [42]. Similarly, the PPV of clinical examination varied according to body region: head (91%) [41], thorax (70–90%) [11, 41], abdomen (43–70%) [41, 43] pelvis (69%) [41], spine (62%) [41], and extremity injuries (91%) [41]. Our study confirmed that the accuracy of initial diagnosis of LLTIs remains moderate to low. However the generally lower PPV achieved in our study may have been due to our methodology. We ensured that the clinical examination was not contaminated by in-hospital information; therefore, the diagnostic accuracy may be closer to the truth.

This data form a fundamental piece of information to describe the need for, and support the development of, decision support systems and diagnostic adjuncts in trauma. Uncertainty is recognized as pervasive in medical decision-making [49]. In trauma, especially at the early or pre-hospital timepoint, uncertainty may be the result of evolving physiology, reduced patient responsiveness (e.g. from head injury or intoxication), or lack of availability of diagnostic adjuncts. Our data reveal the extent to which clinician uncertainty is associated with pre-hospital diagnostic accuracy of injuries. Clinician uncertainty was common when diagnosing LLTIs of the head and torso. When clinicians were uncertain, they had a lower threshold to diagnose an injury, and although more patients were correctly identified (higher sensitivity), and fewer people with the condition were missed (lower FNR), more patients without the condition were incorrectly diagnosed (higher FPR) leading to reduced diagnostic precision. In order to reduce uncertainty, clinicians may consider diagnostic adjuncts, such as point-of-care imaging [50], blood tests [51], telemedicine [42, 52], and clinical decision support systems where possible [53].

Knowing which factors may affect diagnostic accuracy allows individual clinicians and pre-hospital services to develop safeguards and systems to minimize errors. Systematic examination of trauma patients, as suggested by the ATLS curricula, may reduce the number of missed injuries [5]. Some injuries may ‘distract’ the clinician from identifying other injuries, and if the patient’s condition can be explained by the identified injuries, the search for further injuries can become less focused [16, 18]. The association between polytrauma and missed injuries may relate more to human factors (e.g. increased effort from task complexity which constrains performance) than a lack of knowledge or training [54]. Multiple injuries may contribute simultaneously to the same physiological process, which is diagnostically challenging. This study has identified that polytrauma, uncertainty and shock reduce diagnostic accuracy. Awareness of these pitfalls could make clinicians better decision-makers.

### Limitations

This study has several limitations. First, the use of retrospective data from a single centre may predispose to an information bias. However, we used primary source contemporary documentation of the examination findings. Second, a “missed” injury only meant “not documented”, even though it may have been treated appropriately. Some LLTIs may be exacerbated by thorough clinical examination, such as log-rolling spinal injuries or springing a pelvic fracture. For these cases, clinicians should have a high index of suspicion, treat expectantly (e.g. with in-line immobilisation and pelvic binders, respectively), and promptly obtain definitive imaging. Third, although our reference standard used primary data sources, which were corroborated by a national data system, there may be life-threatening physiological injuries not identified by our anatomical reference index (e.g. impact brain apnoea). Fourth, pre-hospital data were derived from written injury assessments, and level of certainty was derived from any documented indication of uncertainty. Clinicians’ diagnostic uncertainty may have existed without being documented, which would have been classified as certain. Fifth, although we evaluated clinical examination, it was not possible to remove the influences of scene assessment, history, and mechanism of injury, which all contribute to diagnosis. Sixth, we assessed the diagnostic accuracy of initial clinical examination when performed by experienced trauma clinicians in an urban pre-hospital setting. These findings may not be directly generalisable to other settings. In particular, if these results are extrapolated to settings where clinicians have less or more experience, the outcomes may differ.

This study also has several strengths. As it assesses diagnostic accuracy of pre-hospital clinical examination, there is very low risk of contamination from in-hospital diagnostic information. Further, the risk that the results are biased by a lack of clinician experience is low, because the study assesses clinical examinations performed by experienced trauma physicians. The study includes a reasonably large absolute number of assessments, which were undertaken consecutively. These results may be applicable to pre-hospital critical care organisations in similar geo-political settings.

## Conclusions

Clinicians must appreciate the limitations of clinical examination—particularly the difficulty in identifying torso haemorrhage—when making clinical decisions in trauma. Clinical examination performed by experienced senior trauma clinicians has only a moderate ability to detect LLTIs. Polytrauma, shock, and diagnostic uncertainty worsen accuracy. Uncertainty improves sensitivity, but worsens positive predictive value, impeding diagnostic precision. The implications of these findings on resuscitative decision-making are wide-reaching, including basing therapeutic decisions on overall risk assessments rather than diagnoses in this uncertain environment, and providing impetus for diagnostic adjuncts and decision support systems in trauma.

## Supplementary Information

**Additional file 1**. Supplementary Tables 1–6.

## Data Availability

The datasets used and/or analysed during the current study are available from the corresponding author on reasonable request, and with permission of Barts Health NHS Trust.

## Abbreviations

LLTIs: Life- and limb-threatening injuries
ATLS: Advanced Trauma Life Support
MTC: Major trauma centre
STARD: Standards for Reporting Diagnostic accuracy studies
LAA: London’s Air Ambulance
AAST: American Association for the Surgery of Trauma
TARN: Trauma Audit and Research Network
AIS: Abbreviated Injury Scale
IQR: Interquartile ranges
PPV: Positive predictive value
NPV: Negative predictive value
FPR: False positive rate
FNR: False negative rate
LR: Likelihood ratio
CI: Confidence interval
MOI: Mechanism of injury
GCS: Glasgow Coma Scale
SBP: Systolic blood pressure
HR: Heart rate
OR: Odds ratio
NCTH: Non-compressible torso haemorrhage
MHP: Major haemorrhage protocol
TXA: Tranexamic acid
