# Supplementary material for: Diagnostic accuracy of clinical examination to identify life- and limb-threatening injuries in trauma patients
Source: Scand J Trauma Resusc Emerg Med. 2023 Apr 7;31:18. doi: 10.1186/s13049-023-01083-z (PMC10082501; doi:10.1186/s13049-023-01083-z)
Supplement: Supplementary file 1 — Additional file 1. Supplementary Tables 1–6. [file 13049_2023_1083_MOESM1_ESM.docx]

# **ADDITIONAL FILE 1**

**LIST OF SUPPLEMENTARY TABLES**

**Supplementary Table 1.** Standards for Reporting Diagnostic accuracy studies (STARD) 2015 checklist.(26)

**Supplementary Table 2.** Diagnostic accuracy of clinical examination to identify life- and limb-threatening injuries and bleeding.

**Supplementary Table 3.** Diagnostic accuracy of clinical examination to identify any injuries, by AIS category.

**Supplementary Table 4.** Pre-hospital trauma clinician assessment of injuries, including suspected injuries and uncertainty, by AIS categories, and by suspected life-and limb-threatening injuries and bleeding, 2019-2020. (n=947)

**Supplementary Table 5.** Diagnostic accuracy of clinical examination to identify life- and limb-threatening injuries and bleeding, when clinicians had high certainty.

**Supplementary Table 6.** Diagnostic accuracy of clinical examination to identify life- and limb-threatening injuries and bleeding, when clinicians were uncertain.

**Supplementary Table 1.** Standards for Reporting Diagnostic accuracy studies (STARD) 2015 checklist.(21)

| **SESSION &** *Topic* | **No** | **Item** | **Reported on page #** |
| --- | --- | --- | --- |
| **TITLE OR ABSTRACT** | **1** | Identification as a study of diagnostic accuracy using at least one measure of accuracy (such as sensitivity, specificity, predictive values, or AUC) | 1-3 Abstract |
| **ABSTRACT** | **2** | Structured summary of study design, methods, results, and conclusions  (for specific guidance, see STARD for Abstracts) | 1-3 Abstract |
| **INTRODUCTION** | **3** | Scientific and clinical background, including the intended use and clinical role of the index test | 4-5 Introduction |
|  | **4** | Study objectives and hypotheses | 4-5 Introduction |
| **METHODS** |  |  |  |
| *Study design* | **5** | Whether data collection was planned before the index test and reference standard were performed (prospective study) or after (retrospective study) | 6 Methods |
| *Participants* | **6** | Eligibility criteria | 6-7 Methods |
|  | **7** | On what basis potentially eligible participants were identified  (such as symptoms, results from previous tests, inclusion in registry) | 6-9 Methods |
|  | **8** | Where and when potentially eligible participants were identified (setting, location and dates) | 6 Methods |
|  | **9** | Whether participants formed a consecutive, random or convenience series | 6 Methods |
| *Test methods* | **10a** | Index test, in sufficient detail to allow replication | 7 Methods |
|  | **10b** | Reference standard, in sufficient detail to allow replication | 7-8 Methods |
|  | **11** | Rationale for choosing the reference standard (if alternatives exist) | 7-8 Methods |
|  | **12a** | Definition of and rationale for test positivity cut-offs or result categories  of the index test, distinguishing pre-specified from exploratory | 7-8 Methods, Table 1 |
|  | **12b** | Definition of and rationale for test positivity cut-offs or result categories  of the reference standard, distinguishing pre-specified from exploratory | 7 Methods |
|  | **13a** | Whether clinical information and reference standard results were available  to the performers/readers of the index test | 7 Methods |
|  | **13b** | Whether clinical information and index test results were available  to the assessors of the reference standard | 6-8 Methods |
| *Analysis* | **14** | Methods for estimating or comparing measures of diagnostic accuracy | 8-9 Methods |
|  | **15** | How indeterminate index test or reference standard results were handled | NA |
|  | **16** | How missing data on the index test and reference standard were handled | NA |
|  | **17** | Any analyses of variability in diagnostic accuracy, distinguishing pre-specified from exploratory | 8-9 Methods |
|  | **18** | Intended sample size and how it was determined | NA |
| **RESULTS** |  |  |  |
| *Participants* | **19** | Flow of participants, using a diagram | 10 Results, no diagram |
|  | **20** | Baseline demographic and clinical characteristics of participants | 10 Results, Table 2 |
|  | **21a** | Distribution of severity of disease in those with the target condition | 10 Results |
|  | **21b** | Distribution of alternative diagnoses in those without the target condition | 10 Results |
|  | **22** | Time interval and any clinical interventions between index test and reference standard | NA |
| *Test results* | **23** | Cross tabulation of the index test results (or their distribution)  by the results of the reference standard | 10-13 Results |
|  | **24** | Estimates of diagnostic accuracy and their precision (such as 95% confidence intervals) | 10-13 Results, Figure 1, Additional File 1 |
|  | **25** | Any adverse events from performing the index test or the reference standard | NA |
| **DISCUSSION** | **26** | Study limitations, including sources of potential bias, statistical uncertainty, and generalisability | 17-18 Limitations |
|  | **27** | Implications for practice, including the intended use and clinical role of the index test | 14-19 Discussion |
| **OTHER INFORMATION** | **28** | Registration number and name of registry | 6 Methods |
|  | **29** | Where the full study protocol can be accessed | NA |
|  | **30** | Sources of funding and other support; role of funders | 21 |

**Supplementary Table 2.** Diagnostic accuracy of clinical examination to identify life- and limb-threatening injuries and bleeding.

| Body region | Injury | Sensitivity, 95% CI | | Specificity, 95% CI | | PPV, 95% CI | | NPV, 95% CI | | FPR, 95% CI | | FNR, 95% CI | | LR |
| --- | --- | --- | --- | --- | --- | --- | --- | --- | --- | --- | --- | --- | --- | --- |
| Head | Traumatic Brain Injury | 69.7 | 63.5 to 75.3 | 84.7 | 81.9 to 87.1 | 59.1 | 53.1 to 64.8 | 89.8 | 87.3 to 91.9 | 15.3 | 12.9 to 18.1 | 30.3 | 24.7 to 36.5 | 4.6 |
| Chest | Chest wall Injury | 62.4 | 52.2 to 71.5 | 93.0 | 91.1 to 94.5 | 49.2 | 40.3 to 58.1 | 95.8 | 94.2 to 96.9 | 7.0 | 5.5 to 8.9 | 37.6 | 28.5 to 47.8 | 8.9 |
|  | Major Hemothorax | 41.0 | 29.5 to 53.5 | 92.9 | 91.0 to 94.4 | 28.4 | 20.0 to 38.6 | 95.8 | 94.3 to 97.0 | 7.1 | 5.6 to 9.0 | 59.0 | 46.5 to 70.5 | 5.8 |
|  | Heart Injury | 40.0 | 71.1 to 76.9 | 99.2 | 98.3 to 99.6 | 20.0 | 3.5 to 51.0 | 99.7 | 99.1 to 99.9 | 0.8 | 0.4 to 1.7 | 60.0 | 23.1 to 92.9 | 47.1 |
|  | Chest Vascular Injury | 31.3 | 14.2 to 55.6 | 90.1 | 88.0 to 91.9 | 5.2 | 2.2 to 11.5 | 98.7 | 97.7 to 99.3 | 9.9 | 8.1 to 12.0 | 68.8 | 44.4 to 85.8 | 3.2 |
|  | Any Chest Bleeding^a^ | 31.9 | 23.2 to 42.0 | 91.0 | 88.8 to 92.8 | 29.0 | 21.0 to 38.5 | 92.1 | 90.0 to 93.8 | 8.1 | 7.2 to 11.2 | 61.3 | 58.0 to 76.8 | 3.6 |
|  | Any Major Chest Injury^b^ | 58.7 | 50.7 to 66.2 | 90.3 | 88.1 to 92.2 | 53.3 | 45.7 to 60.8 | 92.1 | 90.0 to 93.8 | 9.7 | 7.8 to 11.9 | 41.3 | 33.8 to 49.3 | 6.1 |
| Abdomen | Liver Injury | 39.0 | 25.7 to 54.3 | 95.1 | 93.5 to 96.4 | 26.7 | 17.1 to 39.0 | 97.2 | 95.9 to 98.1 | 4.9 | 3.6 to 6.5 | 61.0 | 45.7 to 74.3 | 8.0 |
|  | Spleen Injury | 15.4 | 2.7 to 42.2 | 96.0 | 94.6 to 97.1 | 5.1 | 0.9 to 16.9 | 98.8 | 97.8 to 99.3 | 4.0 | 2.9 to 5.4 | 84.6 | 57.8 to 97.3 | 3.9 |
|  | Renal Injury | 18.2 | 7.3 to 38.5 | 98.1 | 96.9 to 98.8 | 18.2 | 7.3 to 38.5 | 98.1 | 96.9 to 98.8 | 1.9 | 1.2 to 3.1 | 81.8 | 61.5 to 92.7 | 9.3 |
|  | Abdominal Vascular Injury | 7.7 | 3.9 to 33.3 | 97.5 | 96.3 to 98.4 | 4.2 | 0.2 to 20.2 | 98.7 | 97.7 to 99.3 | 2.5 | 1.6 to 3.7 | 92.3 | 66.7 to 99.6 | 3.1 |
|  | Any Abdominal Bleeding^c^ | 42.4 | 31.2 to 54.4 | 89.6 | 87.4 to 91.4 | 23.3 | 16.7 to 31.7 | 95.4 | 93.8 to 96.6 | 10.4 | 8.6 to 12.6 | 57.6 | 45.6 to 68.8 | 4.1 |
|  | Gastrointestinal Injury^d^ | 36.4 | 19.7 to 57.0 | 98.1 | 96.9 to 98.8 | 30.8 | 16.5 to 50.0 | 98.5 | 97.5 to 99.1 | 1.9 | 1.2 to 3.1 | 63.6 | 43.0 to 80.3 | 18.7 |
|  | Any Major Abdominal Injury^e^ | 51.9 | 41.1 to 62.4 | 89.0 | 86.8 to 90.9 | 30.7 | 23.6 to 38.8 | 95.2 | 93.5 to 96.5 | 11.0 | 9.1 to 13.2 | 48.1 | 37.6 to 58.9 | 4.7 |
| Pelvis | Unstable Pelvic Fracture | 23.5 | 12.4 to 40.0 | 99.1 | 98.3 to 99.6 | 50.0 | 28.0 to 72.0 | 97.2 | 95.9 to 98.1 | 0.9 | 0.4 to 1.7 | 76.5 | 60.0 to 87.6 | 26.9 |
| Spine | Unstable Spine Fracture | 14.8 | 59.2 to 32.5 | 94.8 | 93.2 to 96.0 | 7.7 | 3.03 to 18.2 | 97.4 | 96.2 to 98.3 | 5.2 | 4.0 to 6.8 | 85.2 | 67.5 to 94.1 | 2.8 |
|  | Spinal Cord Injury | 45.5 | 21.3 to 72.0 | 99.1 | 98.3 to 99.6 | 38.5 | 17.7 to 64.5 | 99.4 | 98.6 to 99.7 | 0.9 | 0.4 to 1.7 | 54.5 | 28.0 to 78.7 | 53.2 |
|  | Any Spine Injury | 31.4 | 18.6 to 48.0 | 94.6 | 93.0 to 95.9 | 18.3 | 10.6 to 29.9 | 97.3 | 96.0 to 98.2 | 5.4 | 4.1 to 7.0 | 68.6 | 52.0 to 81.4 | 5.9 |
| Extremity | Long Bone Fracture^f^ | 69.9 | 62.3 to 76.6 | 95.3 | 93.6 to 96.6 | 74.3 | 66.6 to 80.7 | 94.3 | 92.4 to 95.7 | 4.7 | 3.4 to 6.4 | 30.1 | 23.4 to 37.7 | 15.0 |
|  | Peripheral Vascular Injury | 30.4 | 15.6 to 50.9 | 98.9 | 98.0 to 99.4 | 41.2 | 21.6 to 64.0 | 98.3 | 97.2 to 98.9 | 1.1 | 0.6 to 2.0 | 69.6 | 49.1 to 84.4 | 28.1 |
|  | Any Extremity Injury | 66.1 | 58.7 to 72.8 | 94.1 | 92.2 to 95.5 | 71.1 | 63.6 to 77.6 | 92.6 | 90.6 to 94.3 | 5.9 | 4.5 to 7.8 | 33.9 | 27.2 to 41.3 | 11.1 |
| Bleeding | Major Chest Bleeding^g^ | 48.1 | 30.7 to 66.0 | 90.5 | 88.5 to 92.3 | 13.0 | 7.8 to 21.0 | 98.3 | 97.2 to 99.0 | 9.5% | 7.7 to 11.5 | 51.9% | 34.0 to 69.3 | 5.09 |
|  | Major Abdominal Bleeding^h^ | 43.6 | 31.4 to 56.7 | 89.2 | 87.0 to 91.1 | 20.0 | 13.8 to 28.0 | 96.3 | 94.7 to 97.3 | 10.8% | 8.9 to 13.0 | 56.4% | 43.3 to 68.6 | 4.05 |
| Supplementary Table 3 Legend: Measures included sensitivity, specificity, positive predictive value (PPV), negative predictive value (NPV), false positive rate (FPR), false negative rate (FNR), and likelihood ratio (LR).  ^a^Any Chest Bleeding includes major hemothorax and chest vascular injury;  ^b^Any Major Chest Injury includes chest wall injury, major hemothorax, heart injury, and chest vascular injury;  ^c^Any Abdominal Bleeding includes liver injury, spleen injury, renal injury, and abdominal vascular injury;  ^d^Gastrointestinal Injury includes stomach, duodenum, jejunum, ileum, colon, rectum, and pancreatico-biliary injuries;  ^e^Any Major Abdominal Injury includes liver, spleen, renal, abdominal vascular, and gastrointestinal injuries;  ^f^Long Bone Fracture includes humerus, radius/ulna, femur and tibia fractures.  ^g^Major Chest Bleeding includes major hemothorax and AAST ≥4 injuries to heart, lung, chest vascular injury  ^h^Major Abdominal Bleeding includes AAST ≥4 injuries to liver, spleen, kidney and abdominal vascular injury | | | | | | | | | | | | | | |

**Supplementary Table 3.** Diagnostic accuracy of clinical examination to identify any injuries, by AIS category.

| Subgroup | AIS category | Sensitivity, 95% CI | | Specificity, 95% CI | | PPV, 95% CI | | NPV, 95% CI | | FPR, 95% CI | | FNR, 95% CI | | LR |
| --- | --- | --- | --- | --- | --- | --- | --- | --- | --- | --- | --- | --- | --- | --- |
| All Injuries | Head | 82.0 | 77.8 to 85.6 | 81.7 | 78.3 to 84.7 | 74.5 | 70.0 to 78.4 | 87.5 | 84.4 to 90.0 | 18.3 | 15.3 to 21.7 | 18.0 | 14.4 to 22.2 | 4.5 |
|  | Face | 68.7 | 62.7 to 74.1 | 85.6 | 82.8 to 88.0 | 63.4 | 57.5 to 68.9 | 88.3 | 85.6 to 90.5 | 14.4 | 12.0 to 17.2 | 31.3 | 25.9 to 37.3 | 4.8 |
|  | Neck | 78.9 | 66.7 to 87.5 | 97.0 | 95.6 to 97.9 | 62.5 | 51.0 to 72.8 | 98.6 | 97.6 to 99.2 | 3.0 | 2.1 to 4.4 | 21.1 | 12.5 to 33.3 | 26.0 |
|  | Chest | 67.2 | 62.4 to 71.6 | 83.2 | 79.8 to 86.1 | 74.9 | 70.2 to 79.1 | 77.2 | 73.7 to 80.4 | 16.8 | 13.9 to 20.2 | 32.8 | 28.4 to 37.6 | 4.0 |
|  | Abdomen | 60.6 | 54.5 to 66.5 | 86.0 | 83.2 to 88.3 | 60.6 | 54.5 to 66.5 | 86.0 | 83.2 to 88.3 | 14.0 | 11.7 to 16.8 | 39.4 | 33.5 to 45.5 | 4.3 |
|  | Pelvis | 46.5 | 36.3 to 57.0 | 94.7 | 92.9 to 96.0 | 46.5 | 36.3 to 57.0 | 94.7 | 92.9 to 96.0 | 5.3 | 4.0 to 7.1 | 53.5 | 43.0 to 63.7 | 8.7 |
|  | Spine | 26.6 | 20.5 to 33.8 | 94.1 | 92.2 to 95.5 | 49.5 | 39.4 to 59.5 | 85.5 | 83.0 to 87.7 | 5.9 | 4.5 to 7.8 | 73.4 | 66.2 to 79.5 | 4.5 |
|  | Upper Limb | 64.6 | 58.9 to 70.0 | 82.6 | 79.5 to 85.3 | 60.9 | 55.3 to 66.3 | 84.8 | 81.8 to 87.3 | 17.4 | 14.7 to 20.5 | 35.4 | 30.0 to 41.1 | 3.7 |
|  | Lower Limb | 86.7 | 82.2 to 90.2 | 77.8 | 74.5 to 80.8 | 61.0 | 56.1 to 65.8 | 93.6 | 91.3 to 95.3 | 22.2 | 19.2 to 25.5 | 13.3 | 9.8 to 17.8 | 3.9 |
|  | Extremity^a^ | 84.7 | 81.1 to 87.8 | 68.1 | 63.9 to 72.1 | 70.2 | 66.2 to 73.9 | 83.4 | 79.5 to 86.7 | 31.9 | 27.9 to 36.1 | 15.3 | 12.2 to 18.9 | 2.7 |
| Certain Injuries | Head | 75.3 | 69.8 to 80.0 | 86.2 | 83.1 to 88.9 | 73.1 | 67.6 to 78.0 | 87.5 | 84.4 to 90.0 | 13.8 | 11.1 to 16.9 | 24.7 | 20.0 to 30.2 | 5.5 |
|  | Face | 65.8 | 59.5 to 71.6 | 86.2 | 83.5 to 88.6 | 61.5 | 55.3 to 67.4 | 88.3 | 85.6 to 90.5 | 13.8 | 11.4 to 16.5 | 34.2 | 28.4 to 40.5 | 4.8 |
|  | Neck | 76.0 | 62.6 to 85.7 | 97.2 | 95.9 to 98.1 | 60.3 | 48.0 to 71.5 | 98.6 | 97.6 to 99.2 | 2.8 | 1.9 to 4.1 | 24.0 | 14.3 to 37.4 | 27.0 |
|  | Chest | 60.6 | 55.3 to 65.7 | 87.1 | 83.9 to 89.7 | 75.2 | 69.7 to 80.0 | 77.4 | 73.8 to 80.6 | 12.9 | 10.3 to 16.1 | 39.4 | 34.3 to 44.7 | 4.7 |
|  | Abdomen | 48.4 | 41.4 to 55.5 | 91.0 | 88.6 to 93.0 | 60.9 | 53.0 to 68.3 | 85.9 | 83.2 to 88.3 | 9.0 | 7.0 to 11.4 | 51.6 | 44.5 to 58.6 | 5.4 |
|  | Pelvis | 37.0 | 26.8 to 48.5 | 98.0 | 96.8 to 98.7 | 61.4 | 46.6 to 74.3 | 94.7 | 92.9 to 96.0 | 2.0 | 1.3 to 3.2 | 63.0 | 51.5 to 73.2 | 18.1 |
|  | Spine | 15.1 | 10.2 to 21.8 | 96.8 | 95.3 to 97.9 | 47.8 | 34.1 to 61.9 | 85.5 | 83.0 to 87.7 | 3.2 | 2.1 to 4.7 | 84.9 | 78.2 to 89.8 | 4.8 |
|  | Upper Limb | 63.3 | 57.4 to 68.9 | 83.7 | 80.7 to 86.4 | 61.5 | 55.7 to 67.0 | 84.8 | 81.8 to 87.3 | 16.3 | 13.6 to 19.3 | 36.7 | 31.1 to 42.6 | 3.9 |
|  | Lower Limb | 85.5 | 80.6 to 89.3 | 79.7 | 76.5 to 82.6 | 61.3 | 56.0 to 66.3 | 93.6 | 91.3 to 95.3 | 20.3 | 17.4 to 23.5 | 14.5 | 10.7 to 19.4 | 4.2 |
|  | Extremity^a^ | 83.1 | 79.1 to 86.4 | 70.2 | 66.0 to 74.1 | 69.7 | 65.5 to 73.7 | 83.4 | 79.5 to 86.7 | 29.8 | 25.9 to 34.0 | 16.9 | 13.6 to 20.9 | 2.8 |
| Uncertain Injuries | Head | 100 | 96.4 to 100 | 0.0 | 0.0 to 11.4 | 77.3 | 69.4 to 83.6 | NA | NA | 100 | 88.6 to 100 | 0.0 | 0.0 to 3.6 | 1.0 |
|  | Face | 100 | 84.5 to 100 | 0.0 | 0.0 to 43.4 | 80.8 | 62.1 to 91.5 | NA | NA | 100 | 56.6 to 100 | 0.0 | 0.0 to 15.5 | 1.0 |
|  | Neck | 100 | 64.6 to 100 | 0.0 | 0.0 to 82.2 | 77.8 | 45.3 to 96.1 | NA | NA | 100 | 17.8 to 100 | 0.0 | 0.0 to 35.4 | 1.0 |
|  | Chest | 98.6 | 92.3 to 99.9 | 0.0 | 0.0 to 13.8 | 74.2 | 64.5 to 82.0 | 0.0 | 0.0 to 94.9 | 100 | 86.2 to 100 | 1.4 | 0.1 to 7.7 | 0.9 |
|  | Abdomen | 100 | 93.9 to 100 | 2.5 | 0.1 to 12.9 | 60.2 | 50.3 to 69.3 | 100 | 5.1 to 1.0 | 97.5 | 87.1 to 99.9 | 0.0 | 0.0 to 6.1 | 1.0 |
|  | Pelvis | 100 | 77.2 to 100 | 0.0 | 0.0 to 11.7 | 31.0 | 19.1 to 46.0 | NA | NA | 100 | 88.3 to 100 | 0.0 | 0.0 to 22.8 | 1.0 |
|  | Spine | 100 | 85.7 to 100 | 0.0 | 0.0 to 14.9 | 51.1 | 37.0 to 65.0 | NA | NA | 100 | 85.1 to 100 | 0.0 | 0.0 to 14.3 | 1.0 |
|  | Upper Limb | 100 | 72.2 to 100 | 0.0 | 0.0 to 29.9 | 52.6 | 31.7 to 72.7 | NA | NA | 100 | 70.1 to 100 | 0.0 | 0.0 to 27.8 | 1.0 |
|  | Lower Limb | 100 | 85.7 to 100 | 0.0 | 0.0 to 19.4 | 59.0 | 43.4 to 72.9 | NA | NA | 100 | 80.6 to 100 | 0.0 | 0.0 to 14.3 | 1.0 |
|  | Extremity^a^ | 100 | 91.8 to 100 | 0.0 | 0.0 to 20.4 | 74.1 | 61.6 to 83.7 | NA | NA | 100 | 79.6 to 100 | 0.0 | 0.0 to 8.2 | 1.0 |
| Supplementary Table 2 Legend: Measures included sensitivity, specificity, positive predictive value (PPV), negative predictive value (NPV), false positive rate (FPR), false negative rate (FNR) and likelihood ratio (LR).  ^a^Extremity includes upper limb or lower limb injury. AIS= abbreviated injury scale; NA= not available because it could not be calculated. | | | | | | | | | | | | | | |

**Supplementary Table 4.** Pre-hospital trauma clinician assessment of injuries, including suspected injuries and uncertainty, by AIS categories, and by suspected life-and limb-threatening injuries and bleeding, 2019-2020. (n=947)

|  | Suspected Injuries  N (%) | Uncertainty Documented  N (%) |
| --- | --- | --- |
| ALL INJURIES | | |
| Head | 411 (43.4) | 132 (32.1) |
| Face | 273 (28.8) | 26 (9.5) |
| Neck | 72 (7.6) | 9 (12.5) |
| Chest | 363 (38.3) | 93 (25.6) |
| Abdomen | 249 (26.3) | 98 (39.4) |
| Pelvis | 86 (9.1) | 42 (48.8) |
| Spine | 91 (9.6) | 45 (49.5) |
| Extremity | 537 (56.7) | 58 (10.8) |
| LIFE- AND LIMB-THREATENING INJURIES AND BLEEDING | | |
| Head |  |  |
| Traumatic Brain Injury | 269 (28.4) | 96 (35.7) |
| Thorax |  |  |
| Chest wall Injury | 118 (12.5) | 35 (29.7) |
| Major Haemothorax | 88 (9.3) | 25 (28.4) |
| Heart Injury | 10 (1.1) | 5 (50.0) |
| Chest Vascular Injury | 97 (10.2) | 26 (26.8) |
| Any Chest Bleeding^a^ | 100 (10.6) | 27 (27.0) |
| Any Major Chest Injury^b^ | 165 (17.4) | 52 (31.5) |
| Abdomen |  |  |
| Liver Injury | 60 (6.3) | 31 (51.7) |
| Spleen Injury | 39 (4.1) | 28 (71.8) |
| Renal Injury | 22 (2.3) | 20 (90.9) |
| Abdominal Vascular Injury | 24 (2.5) | 9 (37.5) |
| Any Abdominal Bleeding^c^ | 120 (12.7) | 76 (63.3) |
| Gastrointestinal Injury^d^ | 26 (2.7) | 5 (19.2) |
| Any Major Abdominal^e^ Injury* | 137 (14.5) | 78 (56.9) |
| Pelvis |  |  |
| Unstable Pelvic Fracture | 16 (1.7) | 4 (25.0) |
| Spine |  |  |
| Unstable Spine Fracture | 52 (5.5) | 30 (57.7) |
| Spinal Cord Injury | 13 (1.4) | 6 (46.2) |
| Any Spine Injury | 60 (6.3) | 34 (56.7) |
| Extremity |  |  |
| Long Bone Fracture^f^ | 144 (15.2) | 12 (8.3) |
| Peripheral Vascular Injury | 17 (1.8) | 6 (35.3) |
| Any Extremity Injury | 159 (16.8) | 19 (11.9) |
| Supplementary Table 4 Legend:  ^a^Any Chest Bleeding includes major haemothorax and chest vascular injury;  ^b^Any Major Chest Injury includes chest wall injury, major haemothorax, heart injury, and chest vascular injury;  ^c^Any Abdominal Bleeding includes liver injury, spleen injury, renal injury, and abdominal vascular injury;  ^d^Gastrointestinal Injury includes stomach, duodenum, jejunum, ileum, colon, rectum, and pancreaticobiliary injuries;  ^e^Any Major Abdominal Injury includes liver, spleen, renal, abdominal vascular, and gastrointestinal injuries;  ^f^Long Bone Fracture includes humerus, radius/ulna, femur and tibia fractures. | | |

**Supplementary Table 5.** Diagnostic accuracy of clinical examination to identify life- and limb-threatening injuries and bleeding, when clinicians had high certainty.

| Body region | Injury | Sensitivity, 95% CI | | Specificity, 95% CI | | PPV, 95% CI | | NPV, 95% CI | | FPR, 95% CI | | FNR, 95% CI | | LR |
| --- | --- | --- | --- | --- | --- | --- | --- | --- | --- | --- | --- | --- | --- | --- |
| Head | Traumatic Brain Injury | 62.4 | 55.2 to 69.2 | 91.0 | 88.6 to 92.9 | 65.3 | 58.0 to 72.0 | 89.9 | 87.4 to 92.0 | 9.0 | 7.1 to 11.4 | 37.6 | 30.8 to 44.8 | 6.9 |
| Chest | Chest wall Injury | 58.0 | 47.2 to 68.2 | 95.7 | 94.0 to 96.8 | 56.6 | 45.9 to 66.8 | 95.9 | 94.3 to 97.0 | 4.3 | 3.2 to 6.0 | 42.0 | 31.8 to 52.8 | 13.4 |
|  | Major Haemothorax | 36.8 | 25.5 to 49.8 | 95.1 | 93.5 to 96.4 | 33.3 | 22.9 to 45.6 | 95.8 | 94.3 to 97.0 | 4.9 | 3.6 to 6.5 | 63.2 | 50.2 to 74.5 | 7.6 |
|  | Heart Injury | 25.0 | 1.3 to 69.9 | 99.6 | 98.9 to 99.8 | 20.0 | 1.0 to 62.4 | 99.7 | 99.1 to 99.9 | 0.4 | 0.2 to 1.1 | 75.0 | 30.1 to 98.7 | 58.6 |
|  | Chest Vascular Injury | 31.3 | 14.2 to 55.6 | 92.7 | 90.8 to 94.2 | 7.0 | 3.1 to 15.4 | 98.7 | 97.7 to 99.3 | 7.3 | 5.8 to 9.2 | 68.8 | 44.4 to 85.8 | 4.3 |
|  | Any Chest Bleeding^a^ | 35.2 | 25.1 to 46.8 | 94.3 | 92.6 to 95.7 | 34.2 | 24.4 to 45.7 | 94.6 | 92.8 to 95.9 | 5.7 | 4.3 to 7.4 | 64.8 | 53.2 to 74.9 | 6.2 |
|  | Any Major Chest Injury^b^ | 53.2 | 44.5 to 61.7 | 94.0 | 92.1 to 95.5 | 59.3 | 50.1 to 67.9 | 92.4 | 90.4 to 94.1 | 6.0 | 4.5 to 7.9 | 46.8 | 38.3 to 55.5 | 8.9 |
| Abdomen | Liver Injury | 30.6 | 18.0 to 46.9 | 98.0 | 96.8 to 98.7 | 37.9 | 22.7 to 56.0 | 97.2 | 95.9 to 98.1 | 2.0 | 1.3 to 3.2 | 69.4 | 53.1 to 82.0 | 14.9 |
|  | Spleen Injury | 0.0 | 0.0 to 25.9 | 98.8 | 97.8 to 99.3 | 0.0 | 0.0 to 25.9 | 98.8 | 97.8 to 99.3 | 1.2 | 0.7 to 2.2 | 100 | 74.1 to 100 | 0 |
|  | Renal Injury | 0.0 | 0.0 to 17.6 | 99.8 | 99.2 to 100 | 0.0 | 0.0 to 82.2 | 98.1 | 96.9 to 98.8 | 0.2 | 0.0 to 0.8 | 100 | 82.4 to 100 | 0 |
|  | Abdominal Vascular Injury | 7.7 | 0.4 to 33.3 | 98.5 | 97.5 to 99.1 | 6.7 | 0.3 to 29.8 | 98.7 | 97.7 to 99.3 | 1.5 | 0.9 to 2.5 | 92.3 | 66.7 to 99.6 | 5.1 |
|  | Any Abdominal Bleeding^c^ | 26.9 | 16.8 to 40.3 | 96.3 | 94.8 to 97.4 | 31.8 | 20.0 to 46.6 | 95.4 | 93.8 to 96.6 | 3.7 | 2.6 to 5.2 | 73.1 | 59.7 to 83.2 | 7.4 |
|  | Gastrointestinal Injury^d^ | 33.3 | 17.2 to 54.6 | 98.5 | 97.5 to 99.1 | 33.3 | 17.2 to 54.6 | 98.5 | 97.5 to 99.1 | 1.5 | 0.9 to 2.5 | 66.7 | 45.4 to 82.8 | 21.9 |
|  | Any Major Abdominal Injury^e^ | 37.1 | 26.2 to 49.5 | 95.5 | 93.9 to 96.8 | 39.0 | 27.6 to 51.7 | 95.2 | 93.5 to 96.5 | 4.5 | 3.2 to 6.1 | 62.9 | 50.5 to 73.8 | 8.3 |
| Pelvis | Unstable Pelvic Fracture | 27.6 | 14.7 to 45.7 | 99.5 | 98.8 to 99.8 | 66.7 | 39.1 to 86.2 | 97.7 | 96.5 to 98.5 | 0.5 | 0.2 to 1.2 | 72.4 | 54.3 to 85.3 | 60.9 |
| Spine | Unstable Spine Fracture | 8.3 | 1.5 to 25.8 | 97.8 | 96.6 to 98.5 | 9.1 | 1.6 to 27.8 | 97.5 | 96.3 to 98.4 | 2.2 | 1.5 to 3.4 | 91.7 | 74.2 to 98.5 | 3.7 |
|  | Spinal Cord Injury | 33.3 | 12.1 to 64.6 | 99.6 | 98.9 to 99.8 | 42.9 | 15.8 to 75.0 | 99.4 | 98.6 to 99.7 | 0.4 | 0.2 to 1.1 | 66.7 | 35.4 to 87.9 | 77.0 |
|  | Any Spine Injury | 24.1 | 12.2 to 42.1 | 97.8 | 96.6 to 98.6 | 26.9 | 13.7 to 46.1 | 97.5 | 96.2 to 98.3 | 2.2 | 1.4 to 3.4 | 75.9 | 57.9 to 87.8 | 11.1 |
| Extremity | Long Bone Fracture^f^ | 68.5 | 60.6 to 75.5 | 95.9 | 94.3 to 97.1 | 75.8 | 67.8 to 82.3 | 94.2 | 92.4 to 95.6 | 4.1 | 2.9 to 5.7 | 31.5 | 24.5 to 39.4 | 16.8 |
|  | Peripheral Vascular Injury | 23.8 | 10.6 to 45.1 | 99.3 | 98.6 to 99.7 | 45.5 | 21.3 to 72.0 | 98.3 | 97.2 to 98.9 | 0.7 | 0.3 to 1.4 | 76.2 | 54.9 to 89.4 | 36.3 |
|  | Any Extremity Injury | 64.2 | 56.6 to 71.2 | 95.2 | 93.5 to 96.5 | 74.3 | 66.5 to 80.8 | 92.5 | 90.5 to 94.2 | 4.8 | 3.5 to 6.5 | 35.8 | 28.8 to 43.4 | 13.5 |
| Bleeding | Major Chest Bleeding^g^ | 41.7 | 24.5 to 61.2 | 93.0 | 91.1 to 94.5 | 13.7 | 7.6 to 23.4 | 98.3 | 97.2 to 99.0 | 7.0% | 5.5 to 8.9 | 58.3% | 38.8 to 75.5 | 5.93 |
|  | Major Abdominal Bleeding^h^ | 27.9 | 16.7 to 42.7 | 96.1 | 94.6 to 97.2 | 27.3 | 16.3 to 41.8 | 96.3 | 94.7 to 97.3 | 3.9% | 2.8 to 5.4 | 72.1% | 57.3 to 83.3 | 7.22 |
| Supplementary Table 5 Legend: Measures included sensitivity, specificity, positive predictive value (PPV), negative predictive value (NPV), false positive rate (FPR), false negative rate (FNR), and likelihood ratio (LR).  ^a^Any Chest Bleeding includes major haemothorax and chest vascular injury;  ^b^Any Major Chest Injury includes chest wall injury, major haemothorax, heart injury, and chest vascular injury;  ^c^Any Abdominal Bleeding includes liver injury, spleen injury, renal injury, and abdominal vascular injury;  ^d^Gastrointestinal Injury includes stomach, duodenum, jejunum, ileum, colon, rectum, and pancreatico-biliary injuries;  ^e^Any Major Abdominal Injury includes liver, spleen, renal, abdominal vascular, and gastrointestinal injuries;  ^f^Long Bone Fracture includes humerus, radius/ulna, femur and tibia fractures.  ^g^Major Chest Bleeding includes major hemothorax and AAST ≥4 injuries to heart, lung, chest vascular injury  ^h^Major Abdominal Bleeding includes AAST ≥4 injuries to liver, spleen, kidney and abdominal vascular injury | | | | | | | | | | | | | | |

**Supplementary Table 6.** Diagnostic accuracy of clinical examination to identify life- and limb-threatening injuries and bleeding, when clinicians were uncertain.

| Body region | Injury | Sensitivity, 95% CI | | Specificity, 95% CI | | PPV, 95% CI | | NPV, 95% CI | | FPR, 95% CI | | FNR, 95% CI | | LR |
| --- | --- | --- | --- | --- | --- | --- | --- | --- | --- | --- | --- | --- | --- | --- |
| Head | Traumatic Brain Injury | 97.9 | 88.9 to 99.9 | 3.9 | 0.7 to 13.0 | 47.9 | 38.2 to 57.8 | 66.7 | 11.8 to 98.3 | 96.2 | 87.0 to 99.3 | 2.1 | 0.1 to 11.1 | 1.0 |
| Chest | Chest wall Injury | 91.7 | 64.6 to 99.6 | 4.0 | 0.2 to 19.5 | 31.4 | 18.6 to 48.0 | 50.0 | 2.6 to 97.4 | 96.0 | 80.5 to 99.8 | 8.3 | 0.4 to 35.4 | 0.9 |
|  | Major Haemothorax | 100 | 51.0 to 100 | 0.0 | 0.0 to 15.5 | 16.0 | 6.4 to 34.7 |  | NA | 100 | 84.5 to 100 | 0.0 | 0.0 to 49.0 | 1.0 |
|  | Heart Injury | 100 | 5.1 to 100 | 0.0 | 0.0 to 49.0 | 20.0 | 1.0 to 62.4 |  | NA | 100 | 51.0 to 100 | 0.0 | 0.0 to 94.9 | 1.0 |
|  | Chest Vascular Injury | NA | NA | 3.7 | 0.2 to 18.3 | 0.0 | 0.0 to 12.9 | 100 | 5.1 to 100 | 96.3 | 81.7 to 99.8 | NA | 100 to 100 |  |
|  | Any Chest Bleeding^a^ | 100 | 51.0 to 100 | 0.0 | 0.0 to 14.3 | 14.8 | 5.9 to 32.5 |  | NA | 100 | 85.7 to 100 | 0.0 | 0.0 to 49.0 | 1.0 |
|  | Any Major Chest Injury^b^ | 100 | 83.9 to 100 | 3.0 | 0.2 to 15.3 | 38.5 | 26.5 to 52.0 | 100 | 5.1 to 100 | 97.0 | 84.7 to 99.9 | 0.0 | 0.0 to 16.1 | 1.0 |
| Abdomen | Liver Injury | 100 | 56.6 to 100 | 0.0 | 0.0 to 12.9 | 16.1 | 7.1 to 32.6 |  | NA | 100 | 87.1 to 100 | 0.0 | 0.0 to 43.4 | 1.0 |
|  | Spleen Injury | 100 | 17.8 to 100 | 0.0 | 0.0 to 12.9 | 7.1 | 1.3 to 22.6 |  | NA | 100 | 87.1 to 100 | 0.0 | 0.0 to 82.2 | 1.0 |
|  | Renal Injury | 100 | 51.0 to 100 | 5.9 | 0.3 to 27.0 | 20.0 | 8.1 to 41.6 | 100 | 5.1 to 100 | 94.1 | 73.0 to 99.7 | 0.0 | 0.0 to 49.0 | 1.1 |
|  | Abdominal Vascular Injury | NA | NA | 0.0 | 0.0 to 29.9 | 0.0 | 0.0 to 29.9 |  | NA | 100 | 70.1 to 100 | NA | 100 to 100 |  |
|  | Any Abdominal Bleeding^c^ | 100 | 78.5 to 100 | 0.0 | 0.0 to 5.8 | 18.4 | 11.3 to 28.6 |  | NA | 100 | 94.2 to 100 | 0.0 | 0.0 to 21.5 | 1.0 |
|  | Gastrointestinal Injury^d^ | 100 | 5.1 to 100 | 0.0 | 0.0 to 49.0 | 20.0 | 1.0 to 62.4 |  | NA | 100 | 51.0 to 100 | 0.0 | 0.0 to 94.9 | 1.0 |
|  | Any Major Abdominal Injury^e^ | 100 | 83.2 to 100 | 0.0 | 0.0 to 6.1 | 24.4 | 16.2 to 34.9 |  | NA | 100 | 93.9 to 100 | 0.0 | 0.0 to 16.8 | 1.0 |
| Pelvis | Unstable Pelvic Fracture | 0.0 | 0.0 to 43.4 | 86.7 | 70.3 to 94.7 | 0.0 | 0.0 to 49.0 | 83.9 | 67.4 to 92.9 | 13.3 | 5.3 to 29.7 | 100 | 56.6 to 100 | 0 |
| Spine | Unstable Spine Fracture | 66.7 | 11.8 to 98.3 | 6.7 | 1.2 to 21.3 | 6.7 | 1.2 to 21.3 | 66.7 | 11.8 to 98.3 | 93.3 | 78.7 to 98.8 | 33.3 | 1.7 to 88.2 | 0.7 |
|  | Spinal Cord Injury | 100 | 17.8 to 100 | 6.7 | 39.1 to 86.2 | 33.3 | 5.9 to 70.0 | 100 | 67.6 to 100 | 33.3 | 13.8 to 60.9 | 0.0 | 0.0 to 82.2 | 3.0 |
|  | Any Spine Injury | 66.7 | 30.0 to 94.1 | 23.1 | 12.6 to 38.3 | 11.8 | 4.7 to 26.6 | 81.8 | 52.3 to 96.8 | 76.9 | 61.7 to 87.4 | 33.3 | 5.9 to 70.0 | 0.9 |
| Extremity | Long Bone Fracture^f^ | 100 | 64.6 to 100 | 54.5 | 28.0 to 78.7 | 58.3 | 32.0 to 80.7 | 100 | 61.0 to 100 | 45.5 | 21.3 to 72.0 | 0.0 | 0.0 to 35.4 | 2.2 |
|  | Peripheral Vascular Injury | 100 | 17.8 to 100 | 60.0 | 31.3 to 83.2 | 33.3 | 5.9 to 70.0 | 100 | 61.0 to 100 | 40.0 | 16.8 to 68.7 | 0.0 | 0.0 to 82.2 | 2.5 |
|  | Any Extremity Injury | 100 | 70.1 to 100 | 52.4 | 32.4 to 71.7 | 47.4 | 27.3 to 68.3 | 100 | 74.1 to 100 | 47.6 | 28.3 to 67.6 | 0.0 | 0.0 to 29.9 | 2.1 |
| Bleeding | Major Chest Bleeding^g^ | 1 | 43.9 to 100 | 0.0 | 0.0 to 13.8 | 11.1 | 3.9 to 28.1 |  | NA | 100 | 86.2 to 100 | 0.0% | 0.0 to 56.1 | 1.0 |
|  | Major Abdominal Bleeding^h^ | 1 | 75.8 to 100 | 0.0 | 0.0 to 5.7 | 15.8 | 9.3 to 25.6 |  | NA | 100 | 94.3 to 100 | 0.0% | 0.0 to 24.2 | 1.0 |
| Supplementary Table 6 Legend: Measures included sensitivity, specificity, positive predictive value (PPV), negative predictive value (NPV), false positive rate (FPR), false negative rate (FNR) and likelihood ratio (LR).  ^a^Any Chest Bleeding includes major haemothorax and chest vascular injury;  ^b^Any Major Chest Injury includes chest wall injury, major haemothorax, heart injury, and chest vascular injury;  ^c^Any Abdominal Bleeding includes liver injury, spleen injury, renal injury, and abdominal vascular injury;  ^d^Gastrointestinal Injury includes stomach, duodenum, jejunum, ileum, colon, rectum, and pancreatico-biliary injuries;  ^e^Any Major Abdominal Injury includes liver, spleen, renal, abdominal vascular, and gastrointestinal injuries;  ^f^Long Bone Fracture includes humerus, radius/ulna, femur and tibia fractures. NA= not available because it could not be calculated.  ^g^Major Chest Bleeding includes major hemothorax and AAST ≥4 injuries to heart, lung, chest vascular injury  ^h^Major Abdominal Bleeding includes AAST ≥4 injuries to liver, spleen, kidney and abdominal vascular injury | | | | | | | | | | | | | | |
